# Weight change during chemotherapy in breast cancer patients: a meta-analysis

**DOI:** 10.1186/s12885-017-3242-4

**Published:** 2017-04-12

**Authors:** M.M.G.A. van den Berg, R.M. Winkels, J.Th.C.M. de Kruif, H.W.M van Laarhoven, M. Visser, J.H.M. de Vries, Y.C. de Vries, E. Kampman

**Affiliations:** 1 Division of Human Nutrition, Wageningen University, Stippeneng 4, 6708 WE Wageningen, The Netherlands; 2 Department of Health Sciences, VU University Amsterdam, De Boelelaan 1085, 1081, HV Amsterdam, The Netherlands; 3 Academic Medical Center, Medical Oncology, Meibergdreef 9, F4-224, 1105 AZ Amsterdam, The Netherlands; 4 Department of Internal Medicine, Nutrition and Dietetics, VU University Medical Center, De Boelelaan 1117, 1007 MB Amsterdam, The Netherlands; 5 Top Institute Food and Nutrition, Nieuwe Kanaal 9A, 6709 PA Wageningen, The Netherlands

**Keywords:** Breast cancer, Chemotherapy, Weight change, Meta-analysis

## Abstract

**Background:**

Weight gain during chemotherapy in women with breast cancer is commonly reported.

However, there are important differences between studies that examined weight change during chemotherapy; e.g. type of chemotherapy, menopausal status, time between body weight measurements and sample size. The purpose of this meta-analysis was to quantify changes in body weight during chemotherapy for women with breast cancer, taking these differences into account.

**Methods:**

We identified relevant studies using PubMed, Scopus and Embase databases. The search was limited to human studies published in English up to and including December 2015. Only studies among women with early stage breast cancer treated with chemotherapy, with reported body weight before and after chemotherapy and type of chemotherapy were included. Random-effect models were used, and heterogeneity between studies was explored through stratified analyses and meta-regression. Sensitivity analyses were done to explore whether a specific study markedly affected the results.

**Results:**

In total 25 papers were found, including data from 2620 women. Overall, body weight increased during chemotherapy: 2.7 kg (95% CI 2.0, 7.5) with a high degree of heterogeneity (I^2^ = 94.2%). Stratified analyses showed weight gain in all strata, but did not substantially reduce heterogeneity. Univariate meta-regression showed less weight gain in prospective studies compared to chart review studies (−2.0, 95% CI: -3.1, −0.8). Studies including cyclophosphamide, methotrexate and 5-fluorouracil (CMF) regimes showed a greater weight gain compared to those that did not (2.2, 95% CI: 1.1, 3.3); and papers published until the year 2000 showed a greater weight gain compared to those published after 2000 (1.9, 95% CI:-0.8, 3.1). In the multivariate models only studies including CMF regimes and studies published until 2000 were associated with significant weight gain of respectively 1.3 and 1.4 kg.

**Conclusion:**

Despite the high heterogeneity, this meta-analysis shows significant weight gain during chemotherapy for women with breast cancer. Weight gain was more pronounced in papers published until 2000 and women receiving CMF as chemotherapy regime. Although weight gain after chemotherapy has decreased over the course of time, weight gain is still substantial and deserves clinical attention.

**Electronic supplementary material:**

The online version of this article (doi:10.1186/s12885-017-3242-4) contains supplementary material, which is available to authorized users.

## Background

Treatment for early stage breast cancer mostly consists of a combination of surgery, radiotherapy, chemotherapy and hormonal therapy. Chemotherapy can cause various side effects, such as nausea, vomiting, hair loss, fatigue, mucositis, cytopenia, ovarian failure and cardiac toxicity. In addition, numerous studies have described weight gain in women with breast cancer during chemotherapy [1–8].

Several reviews reported body weight gain during chemotherapy for breast cancer patients [9–15]. Weight gain during chemotherapy was first reported in 1978 by Dixon et al. [16]. Mid-1990s reviews of the literature suggest that significant weight gain occurred in 50–96% of the breast cancer patients who received chemotherapy. Weight gain was reported to vary from 2.5 to 6.2 kg, while gains of more than 10 kg were not unusual [13, 14, 17]. More recent studies report a lower prevalence of weight gain (35–85%), with weight gain varying between 1.4 to 5.0 kg [6–8, 18–20].

Body weight gain during chemotherapy treatment for breast cancer is undesirable, since it has negative influences on quality of life and health. Weight gain during treatment is associated with an negative affect on quality of life and self-esteem. In addition, several studies reported an increased risk of disease recurrence and poorer prognosis, however, these results are inconsistent [10, 15, 21–25]. A recent meta-analysis concluded that a weight gain of 10% or more after diagnosis of breast cancer is associated with higher all-cause mortality, mainly attributable to 1 study [26].

There are important differences between studies that examined weight change during chemotherapy in breast cancer patients, which may partly explain the large variation in body weight changes observed between studies. First, the amount and type of chemotherapy changed over time, from cyclophosphamide, methotrexate and 5-fluorouracil (CMF) in the 1970s and 1980s, to anthracyclines in the 1990s, to more taxane-based regimens nowadays [27–29]. Second, characteristics of included patients differed between studies. Some studies investigated only premenopausal women, while other studies included both, pre- and postmenopausal women. A third important difference is the time between the body weight measurements. Some studies followed patients only during chemotherapy with body weight measured before and shortly after chemotherapy. Other studies followed patients for a year or even longer with varying moments of weight measurements during follow-up. Fourth, the sample size varied substantially between studies, ranging from less than 10 till more than 200 participants. A fifth important difference is the study design: some studies retrieved body weight as reported in the medical records, while other studies had a prospective design with standardized measurements of body weight before, during and after chemotherapy.

Reviews regarding body weight gain during chemotherapy for breast cancer patients were narrative reviews and did not provide summary estimates for weight change so far. Therefore, the purpose of this meta-analysis was to quantify changes in body weight during chemotherapy for women with early stage breast cancer, and to assess which factors contributed to the heterogeneity between studies.

## Methods

### Literature search

A comprehensive search of literature was conducted using PubMed, Scopus and Embase databases. Search term included: “body weight change”, “body weight”, “breast cancer”, “breast neoplasm”, “breast carcinoma”, “breast tumor”, “breast tumour”, “breast adenoma”, “mamma,” “chemotherapy”, “chemo” and “cytostatic” (see Additional file 1 for more details). The search was limited to human studies, published in English up to and including December 2015. In addition, references listed in papers were screened for additional papers, resulting in the inclusion of one additional paper.

### Paper selection

Papers were included if they met the following criteria: early stage breast cancer patients treated with chemotherapy, type of chemotherapy reported, at least two measurements of body weight: one before and one after chemotherapy treatment. Both observational and intervention studies were included. Intervention studies were included if they included a control group receiving usual care; only the information of this usual care group was included in the meta-analysis.

One database was created and duplicate references were deleted. First, titles were screened on eligibility by two researchers (MB and RW). Secondly, abstracts were screened. If an abstract did not contain sufficient information to assess eligibility, the full-text was reviewed to assess eligibility. Communication letters, abstracts and poster of conferences were excluded.

### Data extraction

From each relevant paper, information on first author, year of publication, country, study design, sample size, characteristics of study population (baseline age, baseline height, baseline menopausal status), breast cancer stage, type of chemotherapy, duration of chemotherapy, follow-up period between measurements of weight, adjuvant/neo adjuvant chemotherapy, time points of weight assessment in relation to start and stop dates of chemotherapy, and weight or weight change (kg) with standard deviation (SD), 95% confidence interval or range were extracted and stored in a database.

### Quality assessment

To assess whether studies of lesser quality could have influenced the results, two researchers (MB and RW) independently assessed the quality of the included studies using an adapted version of the Newcastle-Ottawa Scale for assessing the quality of nonrandomised studies [30]. Studies could get a maximum of 6 points, in four quality areas: 1) representativeness of the sample (information about number of people eligible and included); 2) loss to follow-up of participants (information about number lost to follow-up); 3) information about exposure (type of chemotherapy regimens); 4) assessment of the outcome (information how body weight was assessed). The rating system scores studies from 0 (low quality) to 6 points (high quality). A total score of 3 or less points was considered low quality, whereas a score of 4 or more points was considered high quality.

### Statistical analysis

When no mean body weight change or SE was reported these were calculated if possible for each paper. When data on mean baseline weight and height were available we calculated the baseline mean BMI for the total group of participants using the formula: BMI = weight (kg) /height^2^ (m). If weight or weight change was reported for different types of chemotherapy or menopausal status separately, these results were included instead of the overall mean weight change. Random-effect models were used to calculate the mean and 95% confidence interval of the weight change during chemotherapy for breast cancer. Statistical heterogeneity between studies was assessed by the I^2^ statistic. I^2^ of 25%, 50% or 75% were interpreted as indicating low, moderate and high heterogeneity, respectively [31]. To investigate potential sources of heterogeneity, we conducted stratified analyses. These included the factors: type of chemotherapy (CMF included vs no CMF included), sample size (*n* = <100 vs *n*= > 100), menopausal status (premenopausal, postmenopausal, both), baseline mean BMI (20.0–24.9 vs 25.0–29.9), study design (prospective vs chart review), second measurement of body weight (the end of chemotherapy /6 months after baseline’ group and vs ‘6 months after chemotherapy/12 months after baseline’ group), year of publication (before and including 2000 vs after 2000), country (US, Canada, Western Europe, Australia, Turkey, Korean) and study quality (low quality vs high quality). Of all factors included in the stratified analysis with data available of all estimates we conducted meta-regression analyses. We included the factors that were statistically significant in the univariate stratified analyses in a multivariate regression analysis. Regression coefficients, 95% confidence intervals and *p* values were reported. Sensitivity analyses were conducted by excluding one study at a time to explore whether one study markedly affected the results or highly contributed to the heterogeneity. A second sensitivity analysis was conducted by excluding the only intervention study included. Finally, sensitivity analyses were done excluding studies included <50 participants, and excluding studies included >200 participants to explore whether the smallest or largest studies markedly affect the results. Statistical analyses were conducted using STATA version 11 (StataCorp, College Station, TX). A *p*-value <0.05 was considered statistically significant.

## Results

**Fig. 1:**
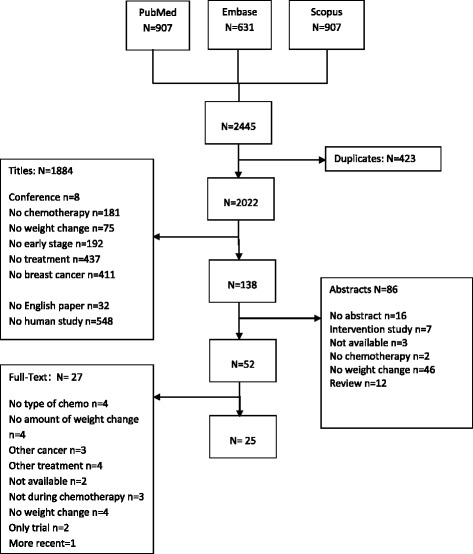
Paper screening and data extraction progress

The results of the literature search and study selection are summarized in Fig. 1. In total the database searches yielded 2445 references. After duplicates were deleted 2022 titles and 138 abstracts were screened for eligibility. A total of 52 full texts were screened, of which 27 papers were excluded, resulting in 25 eligible papers. Papers were excluded for the following reasons: full-text could not be obtained (*n* = 2); articles did not report a weight change (*n* = 4); articles included a variety of cancer types and did not report results for breast cancer separately (*n* = 3); articles did not report weight changes during chemotherapy (*n* = 3); weight change was not reported in kg, but only as percentage change (*n* = 4); type of chemotherapy was not reported (*n* = 4); chemotherapy was combined with other treatment e.g. radiotherapy (*n* = 4); only an intervention group (*n* = 2). One paper was excluded because a more recent paper about the same study was published. In total, 34 weight change estimates from 25 papers were included in this meta-analysis. Six papers reported results for weight gain in subgroups receiving different kind of chemotherapy treatments.

### Characteristics of the participants and study designs

Characteristics of the studies included in this meta-analysis are shown in Table 1. The 25 papers were published between 1985 and the end of 2015. Thirteen weight change estimates were published up to and including 2000 [17, 32–38], and 21 after 2000 [1, 6–8, 12, 18–20, 39–47]. In total, 20 weight change estimates included patients treated with CMF. Sixteen weight estimates retrieved body weight from medical chart review. Eighteen had a prospective design of which one body weight estimate was an intervention study. Sample size of the body weight estimates varied from 8 to 483 participants. All papers used body weight before start of chemotherapy as baseline measure. For the second time point of measurement we created two groups: 1) ‘the end of chemotherapy /6 months after baseline’ group and 2) ‘the 6 months after chemotherapy/12 months after baseline’ group. The first group contained studies for which the second measurement was either directly after chemotherapy or 6 months after diagnosis, the second group all studies for which the second measurement was 6 months after chemotherapy or 12 months after diagnosis.

**Table 1:** Papers included in this meta-analysis of weight change during chemotherapy for women with early stage breast cancer

First author, year of publication	Year of enrolment	Study design	Sample (sample size, key characteristics)	Type of chemotherapy	Follow-up	Mean weight gain in kg (se) in total group	Subgroup analysis Mean weight gain in kg (se)
Foltz, 1985 [32]	UN	Prospective	*n* = 34, pre- and postmenopausal, stage II, adjuvant	CMF	pretreatment - 6 months	2.99 (2.85)	
Heasman, 1985 [33]	1975–1981	Retrospective Chart review	*n* = 237, pre- and postmenopausal, adjuvant	*n* = 46 single agent chemotherapy *n* = 112 CMF *n* = 79 CMF + prednisone	Pre – posttreatment	4.32 (0.23)	Single agent: 2.72 (0.33) CMF: 3.65 (0.32) CMF + prednisone: 6.20 (0.4)
Huntington, 1985 [34]	UN	Retrospective Chart review	*n* = 29, pre- and postmenopausal, adjuvant	*n* = 18 CMF *n* = 11 CMFVP	Pre – posttreatment	4.58 (0.58)	Premenopausal: 7.67 (0.89) Postmenopausal: 2.63 (0.72) Perimenopausal: 4.76 (0.12)
Goodwin, 1988 [35]	1960–1984	Retrospective Chart review	*n* = 193, pre- and postmenopausal, adjuvant	*n* = 113 CMF *n* = 80 CMF + prednison	Pretreatment - 12 months		CMF: 2.51 (0.24) CMF+ prednison 5.55 (0.62)
Demark–Wahnefried, 1997 [13]	1993–1995	Prospective	*n* = 18, premenopausal, adjuvant	*n* = 9 AC *n* = 5 CAF *n* = 1 CMF *n* = 1 CMF + leucovorin *n* = 1 A + CMF *n* = 1 AC + leucovorin	Pre- posttreatment	0 (3.48)	
Aslani, 1999 [36]	UN	Prospective	*n* = 25, pre- and postmenopausal, adjuvant	CMF	Pre- posttreatment	2.35 (0.62)	
Goodwin, 1999 [37]	1989–1996	Prospective	*n* = 176, pre- and postmenopausal, adjuvant	*n* = 128 non-antracyclines *n* = 48 antracyclines	Pretreatment - 12 months	2.5 (0.36)	
Kutynec, 1999	UN	Prospective	*n* = 8, pre- and perimenopausal, adjuvant	AC	Pre- posttreatment	0 (2.85)	
Demark-Wahnefried, 2001 [12]	1995–1999	Prospective	*n* = 36, premenopausal, adjuvant	*n* = 17 doxorubicin regimens *n* = 12 doxorubicin regimens + tamoxifen, *n* = 6 CMF *n* = 1 CMF + tamoxifen	Pretreatment - 6 months	2.2 (0.37)	
McInnes, 2001 [1]	UN	Retrospective Chart review	*n* = 44, pre- and postmenopausal, adjuvant	*n* = 19 CMF - oral *n* = 6 CMF - iv *n* = 2 CAF - oral *n* = 9 CAF - iv *n* = 8 Other	Pretreatment - 6 months	3.4 (0.33)	
Del Rio, 2002 [39]	UN	Prospective	*n* = 30, premenopausal, adjuvant	CMF	Pretreatment - 6 months	2.8 (0.56)	
Lankester, 2002 [40]	1998	Retrospective Chart review	*n* = 100, pre- and postmenopausal, adjuvant + neo-adjuvant	*n* = 69 FEC *n* = 31 CMF	Pretreatment-before last cycle	3.68 (0.4)	
Freedman, 2004 [7]	1999–2001	Prospective	*n* = 20, pre- and posttreatment, adjuvant	*n* = 8 AC *n* = 10 AC + paclitaxel *n* = 2 AC + docetaxel	Pre- posttreatment	−0.83 (0.81)	
Harvie, 2004 [18]	UN	Prospective	*n* = 17, pre- and postmenopausal, adjuvant	*n* = 12 FEC *n* = 5 CMF	Pre- posttreatment	3.3 (1.02)	
Ingram, 2004 [6]	UN	Prospective	*n* = 76, premenopausal, adjuvant	*n* = 39 AC *n* = 33 CEF *n* = 4 CMF	Pre- posttreatment	1.4 (0.39)	AC: 1 (0.34) CEF: 1.5 (0.77) CMF: 5 (1.8)
Kumar, 2004 [41]	UN	Prospective	*n* = 170, pre- and postmenopausal, adjuvant	*n* = 107 CA *n* = 45 CA + taxol *n* = 17 other	Pre- posttreatment	0.4 (1.13)	
Campbell, 2007 [19]	2001–2003	Prospective	*n* = 10, pre- and postmenopausal, adjuavnt	*n* = 5 CEF *n* = 5 AC	Pre- posttreatment	1.98 (5.06)	
Courneya, 2007 [42]	2003–2005	Trial	*n* = 82 pre- and postmenopausal, adjuvant	*n* = 23 FE100C *n* = 20 AC *n* = 8 CE120F *n* = 3 other non-taxanen *n* = 10 TAC *n* = 14 AC-Taxanen *n* = 4 other taxanen	Pre- posttreatment	1.2 (1.71)	
Makari-Judson, 2007 [20]	1997–2002	Retrospective Chart review	*n* = 123, pre- and postmenopausal, adjuvant	AC, AC + taxanen, CAF, Doxorubicin + CMF, CMF or MF *n* = 109 antracycline containing CT *n* = 14 non antracycline	Pretreatment - 12 months	2.6 (0.57)	
Heideman, 2009 [8]	1974–2006	Retrospective	*n* = 31 (CT only), pre- and postmenopausal, adjuvant	AC, EC, CMF, FAC, FEC, other incl herceptin	Pretreatment - 12 months	2.2 (2.07)	
Heideman, 2009 [8]	1974–2006	Retrospective	*n* = 67 (Combined), pre- and postmenopausal, adjuvant	AC, EC, CMF, FAC, FEC, other incl herceptin	Pretreatment - 12 months	2.6 (1.69)	
Biglia, 2010	2007–2008	Prospective	*n* = 34, premenopausal, adjuvant	*n* = 17 FEC *n* = 17 FEC + taxotere	After surgery-end CT	2.07 (0.45)	
Tredan, 2010 [44]	2004–2006	Prospective	*n* = 242, pre- and postmenopausal, adjuvant	*n* = 110 anthracycline without taxane *n* = 156 anthracycline + taxane *n* = 2 taxane (1%)*n* = 4 missing data	Pretreatment – 6 months after CT	0.7 (0.23)	Premenopausal: 1.2 (0.31) Postmenopausal: 0.2 (0.33)
Basaran, 2011 [45]	2003–2004	Retrospective Chart review	*n* = 171, pre- and postmenopausal, adjuvant	*N* = 111 Antracycline based *N* = 55 Antracycline/taxane *N* = 5 No CT	Pre- posttreatment	1.7 (0.94)	
Jeon, 2014 [46]	2005–2010	Retrospective Chart review	*N* = 108, pre- and postmenopausal, adjuvant	TAC	Pre- posttreatment	3.64 (3.7)	
Winkels, 2014 [47]	2001–2010	Retrospective Chart review	*N* = 483, re and postmenopausal	*n* = 289 antracycline based *n* = 170 antracycline + taxane *n* = 10 CMF *n* = 14 other	Pre-posttreatment	1.2 (0.25)	

*UN* unknown

Overall, data from 2620 women were included in this meta-analysis. The mean age of the study samples ranged from 39 to 56 years. Most papers included a combination of pre- and postmenopausal women. Seven papers included only premenopausal women. Two papers showed results separately for pre- and postmenopausal women. Table 2 gives an overview of the quality assessment of the studies included in this meta-analysis. Eight papers scored a total of three or less points for study quality and were assessed as low quality studies.

**Table 2:** Summary of the quality assessment of included studies using an adapted version of the Newcastle-Ottawa scale for assessing the quality of nonrandomised studies

First author, year of publication	Representativeness of sample (2 points)	Loss to follow-up of participants (1 point)	Information about exposure (1 point)	Measurement of outcome (2 points)	Total score
Foltz, 1985 [32]	2	1	1	1	5/6
Heasman, 1985 [33]	2	1	1	1	5/6
Huntington, 1985 [34]	1	0	1	1	3/6
Goodwin, 1988 [35]	2	1	1	1	5/6
Demark-Wahnefried, 1997 [13]	1	1	0	1	3/6
Aslani, 1999 [36]	1	1	1	1	4/6
Goodwin, 1999 [37]	1	1	0	2	4/6
Kutynec, 1999 [38]	2	1	1	1	5/6
Demark-Wahnefried, 2001 [12]	2	1	0	2	5/6
McInnes, 2001 [1]	1	1	0	1	3/6
Del Rio, 2002 [39]	1	1	1	0	3/6
Lankester, 2002 [40]	1	1	0	1	3/6
Freedman, 2004 [7]	1	1	0	2	4/6
Harvie, 2004 [18]	1	1	0	2	4/6
Ingram, 2004 [6]	2	1	1	2	6/6
Kumar, 2004 [41]	2	1	0	1	4/6
Campbell, 2007 [19]	1	1	0	2	4/6
Courneya, 2007 [27]	2	1	0	1	4/6
Makari-Judson, 2007 [20]	2	1	0	1	4/6
Heideman, 2009 [8]	2	1	0	1	4/6
Biglia, 2010	1	1	0	0	2/6
Tredan, 2010 [44]	1	1	0	1	3/6
Basaran, 2011 [45]	1	0	0	1	2/6
Jeon, 2014 [46]	1	1	1	1	4/6
Winkels, 2014 [47]	2	1	1	1	5/6

(1) Representativeness of sample (2 points: extensive information on number of people eligible and included, 1 point: extensive information about recruitment, but not about number of people eligible and included, 0 points: only brief information about recruitment. (2) Loss to follow-up of participants (1 point: information about number lost to follow-up; 0 points: no information about number lost to follow-up). (3) information about exposure (1 point: results are given separate for different chemotherapy regimens, 0 points: results are not separated out for chemotherapy regimens). (4) assessment of the outcome (2 points: measurement protocol for body weight, 1 point: body weight information for chart review or measurement without protocol, 0 points: no information on how body weight was assessed). The rating system scores studies from 0 (low quality) to 6 points (high quality)

### Overall estimate

**Fig. 2:**
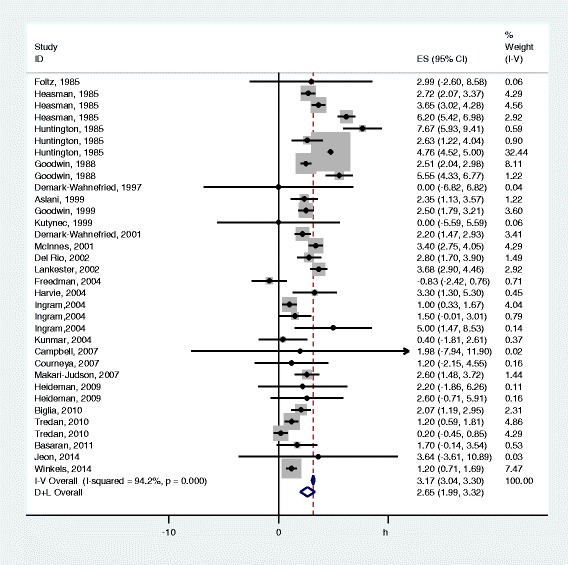
Weight change during chemotherapy for early stage breast cancer. Mean weight changes in individual estimates are depicted as squares with 95% confidence intervals (CI). Pooled estimates with 95% CI are depicted as open diamonds

Mean weight change reported in the papers ranged from −0.8 to 7.7 kg. A gain in body weight was reported in 31 of the 34 estimates, Fig. 2. The pooled mean weight change was 2.7 kg (95% CI: 2.0–3.3) with a heterogeneity of 94.2%. To further explore this high heterogeneity, stratified analyses were conducted.

### Stratified and sensitivity analyses

Body weight change estimates were stratified by type of chemotherapy, sample size, menopausal status, baseline BMI, study design, time between body weight measurements, year of publication, country, and study quality see Table 3. Overall, weight gain was observed in all strata. Stratified analyses did not substantially reduce heterogeneity. The high heterogeneity remained for most subgroups except for the body weight change estimates in studies with a normal mean BMI at baseline (I^2^ = 45.1%) who had a low heterogeneity and estimates not including CMF (I^2^ = 74.7%), including studies with a mean BMI >25 at baseline (I^2^ = 73.2%) and for prospective studies (I^2^ = 69.5%), which all showed a moderate heterogeneity.

**Table 3:** stratified pooled mean weight change and 95% confidence interval in women during chemotherapy treatment for early stage breast cancer

	No of estimates	Pooled weight change kg	95% CI^a^	I^2b^
Overall				
All	34	2.7	2.0, 7.5	94.2
Type Chemotherapy				
CMF included	20	3.5	2.7, 4.3	93.7
No CMF	14	1.4	0.7, 2.0	74.7
Menopausal status				
Premenopausal	9	2.6	1.5, 3.6	86.9
Postmenopausal	2	1.3	−1.1, 3.7	89.4
Perimenopausal	1	4.8	4.5, 5.0	
Combination	22	2.7	2.0, 3.4	88.3
Baseline mean BMI				
20.0–24.9	6	0.5	−0.4; 1.3	45.1
25.0–29.9	15	2.4	1.8; 3.6	73.2
Unknown	13	3.5	2.6; 4.5	95.4
Follow-up				93.8
end of chemotherapy / 6 months after baseline	26	2.7	2.0; 3.5	90.9
6 months after chemotherapy / 12 months after baseline	8	2.4	1.3; 3.4	
Type of study				
Chart review	16	3.6	2.8, 4.4	94.8
Prospective	18	1.6	1.1, 2.2	69.5
Publication year				
Before and including 2000	13	3.8	2.9, 4.7	93.3
After 2000	21	1.9	1.3, 2.5	81.6
Sample Size				
≤ 100	23	3.0	2.2, 3.9	92.7
> 100	11	2.1	1.3, 2.8	90.1
Country				
United States	10	2.8	1.6; 4.1	93.4
Canada	12	3.1	2.1; 4.1	91.8
Western Europe	9	2.0	1.1; 2.8	86.2
Australia	1	2.4	1.1;3.6	
Turkey	1	1.7	−0.1; 3.5	
Korea	1	3.6	−3.6; 10.9	
Study quality				
Low quality	11	2.9	1.6; 4.1	96.7
High quality	23	2.5	1.8; 3.2	88.8

^a^Confidence interval

^b^1^2^ = the percentage heterogeneity due to between-study variation

Sensitivity analyses excluding one study at a time did not markedly influence the overall result of weight change (range 2.4–2.8 kg) nor did importantly affect the amount of heterogeneity (range I^2^ 89.2–94.6%), neither did excluding the smallest or largest studies. In addition, excluding the intervention study did also not markedly influence the overall result of weight change 2.7 kg (95% CI: 2.0–3.4) [42].

Of the 21 body weight change estimates from studies published after 2000, 10 estimates included women treated with CMF. The main weight change in the body weight change estimates from studies after 2000 including women treated with CMF was 2.8 kg (95% CI: 2.0, 3.5) compared to 1.0 kg (95% CI: 0.5, 1.5) in those that did not include women treated with CMF.

### Meta-regression analysis

Results of the meta-regression analyses are shown in Table 4. Results of the univariate model showed that weight gain was significantly different for body weight estimates from studies including CMF vs estimates from studies not including CMF, for studies using chart review vs prospective studies, and for studies published before 2000 vs studies published after 2000. In the multivariate model, we studied the combined effect of type of chemotherapy, study design and year of publication. In this model type of chemotherapy and year of publication remained significantly associated with body weight change, although the body weight change estimates were attenuated. Study design was no longer statistically significantly associated with body weight change in the multivariate model. The residual I^2^ for the multivariable regression model was 84.8%, indicating that these factors explained only a small part of the heterogeneity.

**Table 4:** Results from multivariate meta-regression analysis on weight change in subgroups of early stage breast cancer patients during chemotherapy

	Unadjusted				Adjusted^d^			
	RC^a^	SE^b^	95% CI^c^	*P*-value	RC	SE	95% CI	*P*-value
Type chemotherapy								
No CMF	ref				ref			
CMF included	2.2	0.6	1.1, 3.3	<0.01	1.4	0.6	0.3, 2.6	0.02
Menopausal status								
Premenopausal	ref							
Postmenopausal	−1.3	1.4	−4.1, 1.5	0.36				
Perimenopausal	2.2	1.8	−1.5, 5.8	0.23				
Combination	0.1	0.8	−1.5, 1.6	0.91				
Follow-up								
end of chemotherapy / 6 months after baseline	ref							
6 months after chemotherapy / 12 months after baseline	−0.1	0.1	−0.3; 0.2	0.64				
Type of studie								
Chart review	ref				ref			
Prospective	−2.0	0.6	−3.1, −0.8	<0.01	−0.7	0.6	−1.9, 0.5	0.24
Publication year								
After 2000	ref				ref			
Before and including 2000	1.9	0.6	0.8, 3.1	<0.01	1.3	0.5	0.2, 2.3	0.02
Sample Size								
≤ 100	ref							
> 100	−1.0	0.7	−2.3, 0.4	0.15				
Country								
United States	ref							
Canada	−0.2	0.9	−1.5; 2.0	0.79				
Western Europe	−0.8	0.9	−2.6; 1.0	0.39				
Australia	−0.5	1.9	−4.5; 3.5	0.80				
Turkey	−1.1	2.1	−5.4; 3.1	0.58				
Korea	0.8	4.1	−7.7; 9.3	0.85				
Quality assessment								
Low Quality	ref							
High Quality	−0.4	0.7	−1.8; 1.0	0.58				

^a^Regression coefficient

^b^Standard error

^c^Confidence interval

^d^Adjusted for, type of chemotherapy, type of study and publication year

## Discussion

The present work is the first meta-analysis that quantified changes in body weight during chemotherapy in women with early stage breast cancer. Based on 25 papers, a mean weight gain of 2.7 kg (95% CI: 2.0–3.3) was observed with a heterogeneity of 94.2%. Stratified analysis showed weight gain in all strata, but the strata could only marginally explain the heterogeneity. Adjusted weight gain estimates based on body weight estimates from studies including patients treated with CMF and papers published before 2000 were larger compared to estimates from studies in which CMF was not included and papers published after 2000. Despite the high heterogeneity which could only partly be explained, the results of this meta-analysis suggest constant and significant weight gain during chemotherapy for women with early stage breast cancer.

Treatment for breast cancer has changed over time. Before the 1990s, only CMF was used as chemotherapy regime, while during the 90s the use of anthracyclines gradually increased. In studies after 2004, taxane-based chemotherapy was introduced as a treatment for early stage breast cancer. In the current meta-analysis, CMF emerged as a chemotherapy associated with weight gain, which use has importantly decreased over time. However, our meta-analyses also showed that in studies published after the year 2000 the mean weight gain was still considerable 1.3 kg. Stratified by type of chemotherapy, the mean weight change from studies published after 2000 and including women treated with CMF was 2.8 kg compared to 1.0 kg in those that did not include women treated with CMF. These data suggest that the abandoning of CMF as the chemotherapeutic regimen of choice could be an important reason for observing less weight gain in more recent studies. Independently of CMF, time of publication was associated with weight gain. A possible reason why studies after 2000 observed less weight gain relative to earlier studies could be the incremental use of taxanes in more recent years. However, as the studies included in this meta-analysis did not all provide detailed information on type of chemotherapy, we can only speculate on that.

Another possible explanation for differences in weight gain between older and more recent studies could be age and BMI at baseline. However, we did not see a difference in baseline age and mean BMI comparing older and more recent studies. Yet, since most studies included in this meta-analysis did not provide detailed information and stratified results on baseline BMI, we could not explore this in detail.

Weight gain appeared to be less in prospective studies than in chart review studies in our meta-analysis. A possible explanation for this finding is, that in prospective studies, data usually were collected as part of a cohort or other observational study. These studies could potentially include a selected (e.g. high SES) population, which make them less generalizable to the general population. Chart review papers usually included all patients treated with chemotherapy in a retrospective period of time, but completeness of data was not clearly reported in all studies. Thus both, chart reviews and prospective studies may suffer from incomplete data and selection issues, but as studies did not provide detailed information on response rates and possible selection, we could not explore this further in our meta-analysis. Moreover, stratified results on study quality did not show any differences between studies considered as low quality compared to studies considered of high quality, neither did stratifying on study quality reduce heterogeneity.

An earlier narrative review suggested that women with a normal BMI at baseline were more likely to gain weight compared to women who were overweight at diagnosis [15], however other studies did not confirm this [37, 44] Since only one study included in this meta-analysis reported results for weight change stratified in categories of baseline BMI, we could not study this in great detail. Nevertheless, our analyses suggested a lower weight gain in studies with a mean normal BMI at baseline compared to studies with mean BMI > 25 at baseline. These results should be interpreted carefully, since they represents mean BMI for the total study, which does not mean that all women in that study fall within that BMI category. If the man BMI of a study population is lower, other possible confounding factors may also differ from studies in which mean BMI is higher. However, as this is speculative, and data on other confounding factors is limited, we could not study this further. An important factor in the interpretation of our results is that heterogeneity of our estimates remained high despite elaborate analyses to explore possible sources of heterogeneity, including stratification and meta-regression. This high heterogeneity suggests that other, less studied factors may importantly contribute to weight gain during chemotherapy. A factor that could contribute is ovarian failure which is especially relevant for premenopausal women. This ovarian failure impacts hormonal levels, which may possibly be related to subsequent weight gain. Nevertheless, in the current meta-analyses we did not observe differences in weight gain between pre- and postmenopausal women, possibly because only a small part of the studies stratified for menopausal status [48]. Weight gain may also be explained by common side-effects of chemotherapy such as fatigue, potentially reducing habitual physical activity [17, 49]. Recently, special programs are implemented in breast cancer care in various countries stimulating physical activity. These added interventions may explain differences between older and more recent studies. Also, chemotherapy may induce changes in taste and smell possibly leading to changes in dietary eating patterns which could influence body weight [50]. However, little research has focused on these sensory effects. Furthermore, reductions in energy expenditure in rest have been reported during and after chemotherapy which may lead to an increase in body weight [15, 10]. As most studies did not publish on these potential factors, we could not explore whether they were possible sources of heterogeneity in our meta-analysis.

A limitation of our study is that we could not explore duration of chemotherapy as a source of heterogeneity. Chemotherapy duration has decreased nowadays. Literature suggest that duration of chemotherapy could be an important factor to weight gain [15, 17, 10]. In this meta-analyse it was not possible to explore this since most studies did not report the duration of chemotherapy.

Another limitation is that we used the year that the manuscript was published and not the years the participants were enrolled into the study: time between conducting the study and publishing the results may vary between studies. Although year of enrolment would have been preferable, for 13 estimates this information was available. A sensitivity analysis using only the 21 estimates that had this information available showed a comparable trend of a decrease in weight gain for more recent studies (data not shown).

Also we could only study changes in weight, but not in fat or fat-free mass. Future studies should provide more detailed information on body weight trajectories and preferably body composition, as changes in fat and lean mass may be more clinically relevant. In addition, future studies should also report percentage of women with a significant weight loss, gain or maintenance rather than only mean weight change, so it is possible to establish the clinical magnitude of changes in body weight during chemotherapy.

A strength of this study is that it is, to the best of our knowledge, the first meta-analysis conducted on weight gain in breast cancer women during chemotherapy. A comprehensive literature search was conducted including an additional hand search. This makes the potential of missing any published data in English unlikely.

## Conclusions

In conclusion, our results indicate that women generally gain weight during chemotherapy for early stage breast cancer. This weight gain is more pronounced in women treated with CMF and is greater in studies published before 2000. Although weight gain after chemotherapy has decreased over the course of time, weight gain is still substantial and deserves clinical attention.

## Additional file

Additional file 1: Search Strategy. (DOCX 17 kb)

## Abbreviations

CI: Confidence interval
CMF: Cyclophosphamide, methotrexate and 5-fluorouracil
SD: Standard deviation

## References

[1] McInnes JA, Knobf MT (2001). Weight gain and quality of life in women treated with adjuvant chemotherapy for early-stage breast cancer. Oncol Nurs Forum.

[2] Costa LJ, Varella PC, del Giglio A (2002). Weight changes during chemotherapy for breast cancer. Sao Paulo Med J.

[3] Cheney CL, Mahloch J, Freeny P (1997). Computerized tomography assessment of women with weight changes associated with adjuvant treatment for breast cancer. Am J Clin Nutr.

[4] Goodwin PJ. Obesity and insulin resistance in breast cancer: Are clinical trials needed? Breast Dis. 2012;23:310–13. ISSN: 1043-321X.

[5] Rock CL, Demark-Wahnefried W (2002). Nutrition and survival after the diagnosis of breast cancer: a review of the evidence. J Clin Oncol.

[6] Ingram C, Brown JK (2004). Patterns of weight and body composition change in premenopausal women with early stage breast cancer: has weight gain been overestimated?. Cancer Nurs.

[7] Freedman RJ, Aziz N, Albanes D, Hartman T, Danforth D, Hill S, Sebring N, Reynolds JC, Yanovski JA (2004). Weight and body composition changes during and after adjuvant chemotherapy in women with breast cancer. J Clin Endocrinol Metab.

[8] Heideman WH, Russell NS, Gundy C, Rookus MA, Voskuil DW (2009). The frequency, magnitude and timing of post-diagnosis body weight gain in Dutch breast cancer survivors. Eur J Cancer.

[9] Gadéa E, Thivat E, Planchat E, Morio B, Durando X (2012). Importance of metabolic changes induced by chemotherapy on prognosis of early-stage breast cancer patients: a review of potential mechanisms. Obes Rev.

[10] Vance V, Mourtzakis M, McCargar L, Hanning R (2011). Weight gain in breast cancer survivors: prevalence, pattern and health consequences. Obes Rev.

[11] Visovsky C (2006). Muscle strength, body composition, and physical activity in women receiving chemotherapy for breast cancer. Integr Cancer Ther.

[12] Demark-Wahnefried W, Peterson BL, Winer EP, Marks L, Aziz N, Marcom PK, Blackwell K, Rimer BK (2001). Changes in weight, body composition, and factors influencing energy balance among premenopausal breast cancer patients receiving adjuvant chemotherapy. J Clin Oncol.

[13] Demark-Wahnefried W, Rimer BK, Winer EP (1997). Weight gain in women diagnosed with breast cancer. J Am Diet Assoc.

[14] Demark-Wahnefried W, Winer EP, Rimer BK (1993). Why women gain weight with adjuvant chemotherapy for breast cancer. J Clin Oncol.

[15] Makari-Judson G, Braun B, Jerry DJ, Mertens WC (2014). Weight gain following breast cancer diagnosis: implication and proposed mechanisms. World J Clin Oncol.

[16] Dixon Jk, Moritz DA, Baker FL: Breast cancer and weight gain: an unexpected finding. Oncol. Nurs. Forum. 1978;5:5–7. 248815

[17] Demark-Wahnefried W, Hars V, Conaway MR, Havlin K, Rimer BK, McElveen G, Winer EP (1997). Reduced rates of metabolism and decreased physical activity in breast cancer patients receiving adjuvant chemotherapy. Am J Clin Nutr.

[18] Harvie MN, Campbell IT, Baildam A, Howell A (2004). Energy balance in early breast cancer patients receiving adjuvant chemotherapy. Breast Cancer Res Treat.

[19] Campbell KL, Lane K, Martin AD, Gelmon KA, McKenzie DC (2007). Resting energy expenditure and body mass changes in women during adjuvant chemotherapy for breast cancer. Cancer Nurs.

[20] Makari-Judson G, Judson CH, Mertens WC (2007). Longitudinal patterns of weight gain after breast cancer diagnosis: observations beyond the first year. Breast J.

[21] Thivat E, Therondel S, Lapirot O, Abrial C, Gimbergues P, Gadea E, Planchat E, Kwiatkowski F, Mouret-Reynier MA, Chollet P, Durando X (2010). Weight change during chemotherapy changes the prognosis in non metastatic breast cancer for the worse. BMC Cancer.

[22] Chlebowski RT, Aiello E, McTiernan A (2002). Weight loss in breast cancer patient management. J Clin Oncol.

[23] Bradshaw PT, Ibrahim JG, Stevens J, Cleveland R, Abrahamson PE, Satia JA, Teitelbaum SL, Neugut AI, Gammon MD (2012). Postdiagnosis change in bodyweight and survival after breast cancer diagnosis. Epidemiology.

[24] Caan BJ, Kwan ML, Shu XO, Pierce JP, Patterson RE, Nechuta SJ, Poole EM, Kroenke CH, Weltzien EK, Flatt SW (2012). Weight change and survival after breast cancer in the after breast cancer pooling project. Cancer Epidemiol Biomark Prev.

[25] Camoriano JK, Loprinzi CL, Ingle JN, Therneau TM, Krook JE, Veeder MH (1990). Weight change in women treated with adjuvant therapy or observed following mastectomy for node-positive breast cancer. J Clin Oncol.

[26] Playdon MC, Bracken MB, Sanft TB, Ligibel JA, Harrigan M, Irwin ML (2015). Weight gain after breast cancer diagnosis and all-cause mortality: systematic review and meta-analysis. J Natl Cancer Inst.

[27] Bonadonna G, Valagussa P Fau-Moliterni A, Moliterni A Fau-Zambetti M, Zambetti M Fau-Brambilla C, Brambilla C: Adjuvant cyclophosphamide, methotrexate, and fluorouracil in node-positive breast cancer: the results of 20 years of follow-up. N. Engl. J. Med. 1995;332(14):901–06. 10.1056/NEJM199504063321401 7877646

[28] Lopez-Tarruella S, Martin M (2009). Recent advances in systemic therapy: advances in adjuvant systemic chemotherapy of early breast cancer.

[29] Giordano SH, Lin YL, Kuo YF, Hortobagyi GN, Goodwin JS (2012). Decline in the use of anthracyclines for breast cancer. J Clin Oncol.

[30] GA Wells, B Shea, D O'Connell, J Peterson, V Welch, M Losos, P Tugwell: The Newcastle-Ottawa Scale (NOS) for assessing the quality of nonrandomised studies in meta-analyses. http://www.ohri.ca/programs/clinical_epidemiology/oxford.asp

[31] Higgins JP, Thompson SG, Deeks JJ, Altman DG (2003). Measuring inconsistency in meta-analyses. BMJ.

[32] Foltz AT (1985). Weight gain among stage II breast cancer patients: a study of five factors. Oncol Nurs Forum.

[33] Heasman KZ, Sutherland HJ, Campbell JA, Elhakim T, Boyd NF (1985). Weight gain during adjuvant chemotherapy for breast cancer. Breast Cancer Res Treat.

[34] Huntington MO (1985). Weight gain in patients receiving adjuvant chemotherapy for carcinoma of the breast. Cancer.

[35] Goodwin PJ, Panzarella T, Boyd NF (1988). Weight gain in women with localized breast cancer--a descriptive study. Breast Cancer Res Treat.

[36] Aslani A, Smith RC, Allen BJ, Pavlakis N, Levi JA (1999). Changes in body composition during breast cancer chemotherapy with the CMF-regimen. Breast Cancer Res Treat.

[37] Goodwin PJ, Ennis M, Pritchard KI, McCready D, Koo J, Sidlofsky S, Trudeau M, Hood N, Redwood S (1999). Adjuvant treatment and onset of menopause predict weight gain after breast cancer diagnosis. J Clin Oncol.

[38] Kutynec CL, McCargar L, Barr SI, Hislop TG (1999). Energy balance in women with breast cancer during adjuvant treatment. J Am Diet Assoc.

[39] Del Rio G, Zironi S, Valeriani L, Menozzi R, Bondi M, Bertolini M, Piccinini L, Banzi MC, Federico M (2002). Weight gain in women with breast cancer treated with adjuvant cyclophosphomide, methotrexate and 5-fluorouracil. Analysis of resting energy expenditure and body composition. Breast Cancer Res Treat.

[40] Lankester KJ, Phillips JE, Lawton PA (2002). Weight gain during adjuvant and neoadjuvant chemotherapy for breast cancer: an audit of 100 women receiving FEC or CMF chemotherapy. Clin Oncol (R Coll Radiol).

[41] Kumar N, Allen KA, Riccardi D, Bercu BB, Cantor A, Minton S, Balducci L, Jacobsen PB (2004). Fatigue, weight gain, lethargy and amenorrhea in breast cancer patients on chemotherapy: is subclinical hypothyroidism the culprit?. Breast Cancer Res Treat.

[42] Courneya KS, Segal RJ, Mackey JR, Gelmon K, Reid RD, Friedenreich CM, Ladha AB, Proulx C, Vallance JKH, Lane K (2007). Effects of aerobic and resistance exercise in breast cancer patients receiving adjuvant chemotherapy: a multicenter randomized controlled trial. J Clin Oncol.

[43] Biglia N, Cozzarella M, Ponzone R, Marenco D, Maggiorotto F, Fuso L, Sismondi P (2004). Personal use of HRT by postmenopausal women doctors and doctors’ wives in the north of Italy. Gynecol Endocrinol.

[44] Tredan O, Bajard A, Meunier A, Roux P, Fiorletta I, Gargi T, Bachelot T, Guastalla JP, Lallemand Y, Faure C (2010). Body weight change in women receiving adjuvant chemotherapy for breast cancer: a French prospective study. Clin Nutr.

[45] Basaran G, Turhal NS, Cabuk D, Yurt N, Yurtseven G, Gumus M, Teomete M, Dane F, Yumuk PF (2011). Weight gain after adjuvant chemotherapy in patients with early breast cancer in Istanbul Turkey. Med Oncol.

[46] Jeon YW, Lim ST, Choi HJ, Suh YJ (2014). Weight change and its impact on prognosis after adjuvant TAC (docetaxel-doxorubicin-cyclophosphamide) chemotherapy in Korean women with node-positive breast cancer. Med Oncol.

[47] Winkels RM, Beijer S, van Lieshout R, van Barneveld D, Hofstede J, Kuiper J, Vreugdenhil A, van Warmerdam LJC, Schep G, Blaisse R (2014). Changes in body weight during various types of chemotherapy in breast cancer patients. e-SPEN J.

[48] Gu K, Chen X, Zheng Y, Chen Z, Zheng W, Lu W, Shu XO (2010). Weight change patterns among breast cancer survivors: results from the shanghai breast cancer survival study. Cancer Causes Control.

[49] Irwin ML, Crumley D, McTiernan A, Bernstein L, Baumgartner R, Gilliland FD, Kriska A, Ballard-Barbash R (2003). Physical activity levels before and after a diagnosis of breast carcinoma: the health, eating, activity, and lifestyle (HEAL) study. Cancer.

[50] Boltong A, Aranda S, Keast R, Wynne R, Francis PA, Chirgwin J, Gough K (2014). A prospective cohort study of the effects of adjuvant breast cancer chemotherapy on taste function, food liking, appetite and associated nutritional outcomes. PLoS One.

