# Supplementary material for: Weight change during chemotherapy in breast cancer patients: a meta-analysis
Source: BMC Cancer. 2017 Apr 12;17:259. doi: 10.1186/s12885-017-3242-4 (PMC5389147; doi:10.1186/s12885-017-3242-4)
Supplement: Additional file 1: — Search Strategy. (DOCX 17 kb) [file 12885_2017_3242_MOESM1_ESM.docx]

**SUPPLEMENT 1: Search Strategy**

PUBMED

Weight changes

Body weight change [mesh] OR body weight changes [mesh] OR body weight [tiab] OR weight* [tiab]

Breast cancer

breast cancer [mesh] OR breast cancer [tiab] OR malign* [tiab] OR neoplasm* [tiab] OR carcinoma* [tiab] OR cancer* [tiab] OR tumor* [tiab] OR tumour* [tiab] AND breast* [tiab] or mamma* [tiab]

Chemotherapy

chemotherapy [mesh] OR chemo* [tiab] OR cytostatic [mesh] OR cytostatic* [tiab]

SCOPUS

Weight change

( TITLE-ABS-KEY ( body  weight  change* )  OR  TITLE-ABS-KEY ( body  weight* )  OR  TITLE-ABS-KEY ( weight  change* ) )

Breast caner

( ( TITLE-ABS-KEY ( malign* )  OR  TITLE-ABS-KEY ( neoplasm* )  OR  TITLE-ABS-KEY ( carcinoma* )  OR  TITLE-ABS-KEY ( cancer* )  OR  TITLE-ABS-KEY ( tumor* )  OR  TITLE-ABS-KEY ( tumour* ) ) )  AND  ( ( TITLE-ABS-KEY ( breast* )  OR  TITLE-ABS-KEY ( mamma* ) ) )

Chemotherapy

(TITLE-ABS-KEY(chemo*) OR TITLE-ABS-KEY(cytostatic*))

EMBASE

Weight change

Exp ‘body weight change’/ OR body weight changes. ti,ab OR body weight change*. ti,ab OR body weight. ti,ab

Breast cancer

exp ‘breast cancer’/ OR breast cancer. ti,ab OR malign*. ti,ab OR neoplasm*. ti,ab OR carcinoma*. ti,ab OR cancer*. ti,ab OR tumor*. ti,ab OR tumour*. ti,ab AND breast*. ti,ab OR mamma*.

Chemotherapy

exp ‘chemotherapy/ OR chemo*. ti,ab OR exp cytostatics/ OR cytostatic*. ti,ab
